# Predicting Type 2 Diabetes Mellitus Occurrence Using Three-Dimensional Anthropometric Body Surface Scanning Measurements: A Prospective Cohort Study

**DOI:** 10.1155/2018/6742384

**Published:** 2018-07-08

**Authors:** Ming-Kuo Ting, Pei-Ju Liao, I-Wen Wu, Shuo-Wei Chen, Ning-I Yang, Tzu-Yu Lin, Kuang-Hung Hsu

**Affiliations:** ^1^Division of Endocrinology and Metabolism, Chang Gung Memorial Hospital, Keelung, Taiwan; ^2^Department of Health Care Administration, Oriental Institute of Technology, New Taipei City, Taiwan; ^3^Division of Nephrology, Chang Gung Memorial Hospital, Keelung, Taiwan; ^4^Division of Gastroenterology and Hepatology, Chang Gung Memorial Hospital, Keelung, Taiwan; ^5^Division of Cardiology, Chang Gung Memorial Hospital, Keelung, Taiwan; ^6^Healthy Aging Research Center, Chang Gung University, Taoyuan, Taiwan; ^7^Laboratory for Epidemiology, Department of Health Care Management, Chang Gung University, Taoyuan, Taiwan; ^8^Department of Emergency Medicine, Chang Gung Memorial Hospital, Taoyuan, Taiwan; ^9^Department of Urology, Chang Gung Memorial Hospital, Taoyuan, Taiwan

## Abstract

**Background:**

An accurate and comprehensive anthropometric measure for predicting type 2 diabetes mellitus (T2DM) has not yet been depicted.

**Methods:**

A total of 8450 nondiabetic participants were recruited during 2000–2010 in Taiwan. The cohort was followed up to the end of 2013, over an average of 8.87 years. At recruitment, participants completed a questionnaire related to basic demographics, lifestyle variables, personal disease history, and family disease history. 3D body surface scanning was used to obtain 35 anatomical measurements. A Cox proportional hazard model was used to conduct multivariable analyses.

**Results:**

A total of 2068 T2DM cases at an incidence rate of 27.59 × 10^−3^ (year^−1^) were identified during the follow-up period. Multivariable-adjusted hazard ratios (HRs) demonstrated that neck circumference (NC) (HR = 1.048; 95% CI = 1.033–1.064), waist width (WW) (HR = 1.061; 95% CI = 1.040–1.081), and left thigh circumference (TC) (HR = 0.984; 95% CI = 0.972–0.995) were significant predictors of the occurrence of T2DM. While dividing body measurement into median high/low groups, an increased risk of T2DM was observed among participants with a larger NC and smaller TC (HR = 1.375; 95% CI = 1.180–1.601) and a larger WW and smaller TC (HR = 1.278; 95% CI = 1.085–1.505) relative to other participants.

**Conclusions:**

This study suggests that as well as using traditional waist and TC measurements, NC can be used as an indicator to provide an early prediction of developing T2DM, while providing clues for future mechanistic investigations of T2DM.

## 1. Introduction

Type 2 diabetes mellitus (T2DM) is currently the fastest growing chronic disease in the world. According to WHO calculations, the number of people affected by diabetes will have increased 114% by 2030 relative to the year 2000 [1], and Asia is the region where diabetes is growing the fastest [1, 2]. Chronic complications from the disease result in major health care burdens and affect patient longevity and quality of life. Therefore, knowledge of early markers to predict the disease and the implementation of associated preventive strategies is vital for ameliorating this global problem.

Literature indicates that groups at a high risk for T2DM have a family history of the disease, are overweight or obese, do less exercise, have metabolic syndrome, or have had gestational diabetes mellitus [3]. People with T2DM are at risk of hypertension, hyperlipidemia, and cardiovascular disease. Central obesity, or abdominal visceral fat accumulation, is a major body shape phenotype used to predict the risk of T2DM and is related to an inflammatory response mechanism and insulin resistance [4]. One meta-analysis indicated that body mass index, waist circumference (WC), and waist/hip ratio are three major obesity markers associated with the incidence of T2DM [5]. Another meta-analysis indicated that the use of the waist-to-height ratio (WHtR) may also predict several cardiometabolic risk factors, particularly in diabetes, and is superior to the use of the BMI [6]. Furthermore, other studies have shown that four anthropometric indices (BMI, WC, W/HR, and WHtR) are markers of obesity and may predict the future onset of diabetes, and a number of studies have documented the wide use of WC measurements to approximate abdominal visceral fat accumulation, which is documented as being associated with insulin resistance and cardiometabolic risk [7–10]. However, the neck circumference (NC) has also been used as an alternative measure of upper-body subcutaneous fat, which correlates with whole-body adiposity and abdominal adiposity [7–9, 11]. Taking NC measurements has been demonstrated as practical in a relatively large population [12–14] and is a better predictor of metabolic syndrome than WC [15]. Moreover, the thigh circumference (TC) or waist-to-thigh ratio is reported to be associated with T2DM [16–18], and low subcutaneous thigh fat has been documented as a risk factor for hyperlipidemia and hyperglycemia [19].

Previous literature has demonstrated the use of noninvasive three-dimensional (3D) scanning technology to cross-sectionally associate body measurements, such as waist-to-thigh, with T2DM [18, 20] but no longitudinal studies with long-term follow-up have been performed to date. Therefore, this study investigated selected 3D body measurements in the evaluation of HRs for developing T2DM during an average 8.87-year follow-up in an established cohort.

## 2. Methods

### 2.1. Study Samples

A total of 8450 participants (4431 men and 4019 women) without diabetes were recruited from the Department of Health Promotion and Examination of Chang Gung Memorial Hospital in Taiwan. The study follow-up period spanned from February 2000 to December 2013 (a total of 13 years and 11 months), and the average follow-up period was 8.87 years. This study was approved by the Institutional Review Board of Chang Gung Medical Foundation (97-2538 B).

### 2.2. Anthropometrical Parameters

3D body surface measurements were collected by using whole-body 3D laser scanning according to previously published methods [20, 21]. In addition to body height, body weight, and BMI, 35 measurements from four anatomical regions were found. The trunk region included the chest profile area, chest circumference, chest width, waist profile area, WC, waist width (WW), trunk volume, and trunk surface area. The head and neck region included the head volume, head surface area, head circumference, and NC. The hip to the lower limbs included the hip profile area, hip circumference, hip width, left leg volume, left leg surface area, left calf circumference, right leg volume, right leg surface area, right calf circumference, left TC, right TC, left leg length, and right leg length. The upper limb region included the left arm volume, left arm surface area, left arm length, right arm volume, right arm surface area, right arm length, left upper arm circumference, right upper arm circumference, left forearm circumference, and right forearm circumference. In addition to whole measures of the trunk, the trunk dimension was further classified into chest and waist measures due to the distinguishably anatomic and physiological characteristics. The 3D laser scanning machine (LT3DCam) was built by Logistic Technology Company (LTC, Hsinchu, Taiwan) and was proven with high standard of accuracy due to the objective and comprehensive ways of measuring human body surface. The standard procedure of measuring is to require a subject to remove all outer clothes except for underwear in preparation for scanning (women with bras in addition to pants) and to stand still on the stage for scanning (a total scanning time is about 10 seconds) [19]. The software system will collect, realign, construct, and measure a subject's whole-body digital stature and selected information. The measurement error of the 3D scanner in measuring human surface body has been checked, and the error in the *x*- and *y*-axis is approximately 1 mm (1.2%), and in the *z*-axis it is less than 0.1 mm (0.2%) [22].

### 2.3. Data Collection

Upon recruitment, a questionnaire was administered that requested the following information: date of birth; sex; education; marital status; occupation; history of cigarette smoking, alcohol drinking, and betel nut chewing; personal history of disease (including diabetes, hypertension, heart disease, chronic kidney disease, liver cirrhosis, and chronic hepatitis); and family history of T2DM. A medical chart review confirmed the answers provided. For those with no history of diabetes, a fasting blood glucose level was obtained. Diabetes was defined according to ADA guidelines. For those without a history of hypertension, blood pressure was measured with a mercury sphygmomanometer on the left arm after the patient had been resting for 20 minutes in a seated position. Hypertension was defined according to the guidelines of the Joint National Committee on Detection, Evaluation, and Treatment of High Blood Pressure (systolic blood pressure ≥ 140 mmHg, diastolic blood pressure ≥ 90 mmHg, or the use of antihypertensive medication) [23]. Heart disease was based on medical chart findings and defined using the ICD-9 Clinical Modification (ICD-9-CM) codes 390–398, 410–141, and 420–429. Renal disease, liver cirrhosis, and chronic hepatitis were coded as ICD-9-CM 580–583, ICD-9-CM 571.2 and 571.5, and ICD-9-CM 571.4, respectively. The occurrence of T2DM was determined using the national registration database and then further confirmed with insurance claim data. Patients diagnosed as ICD-9-CM code 250.00 and administered with DM-related drug prescriptions at least three times during the first diagnosis year were identified.

### 2.4. Statistical Analyses

Two independent sample *t*-tests were used to compare differences between the continuous variables of the groups, and results were presented as the mean ± SD. The *χ* ^2^ test was used to differentiate between the distribution of categorical variables, and results were expressed using frequencies and percentages between the groups. 3D body surface measurements were screened using a two-sample *t*-test by comparing differences between patients and controls. To avoid collinearity in the regression analysis, one body measurement with the lowest *p* value was selected from each anatomic dimension for subsequent multivariable analysis. A Cox proportional hazard model was used to determine the strength of the association between the selected body measurements and the incidence of T2DM. In addition to the forced-in sociodemographic variables, a backward model selection with *p* < 0.1 was used to determine variables, including lifestyles, body measures, and underlying diseases, to be retained in the regression model. Results were expressed as hazard ratios (HRs) adjusted for confounding variables, including age, sex, education, marital status, occupation, betel nut chewing, and hypertension history. For stratified analysis, selected body measurements were further categorized into high and low levels, according to the median result. A combination of two body measurements was generated to examine high-risk body shape characteristics. The statistical software used for the analyses in this study was SPSS 22.0 (IBM Corporation, Armonk, NY).

## 3. Results

Out of the 8450 participants (a total of 74964.5 person-years with an average of 8.87 years ranging from 0 to 13.80 years) in this study, a total of 2068 cases of T2DM were identified during the follow-up period, with an incidence rate of 0.02759 per year (Table 1). An increased incidence of disease occurrence was found for participants older than 50.7 years (median) (HR 1.52; 95% CI = 1.36–1.70) compared with younger participants. The study sample consisted of 4431 (52.4%) men and 4019 (47.6%) women who were at risk for developing T2DM. Results showed that participants who had a lower level of education were at a higher risk of developing T2DM. T2DM occurrence was associated with occupational categories: farmers and housekeeping and others were at the highest risk. Among lifestyle variables, betel nut chewers had a higher risk of developing T2DM (HR = 1.26; 95% CI = 1.08–1.47). In addition, participants with a personal disease history of hypertension (HR = 1.98; 95% CI = 1.79–2.18) and heart disease (HR = 1.39; 95% CI = 1.202–1.611) were more likely to develop T2DM (Table 1).

**Figure 1:**
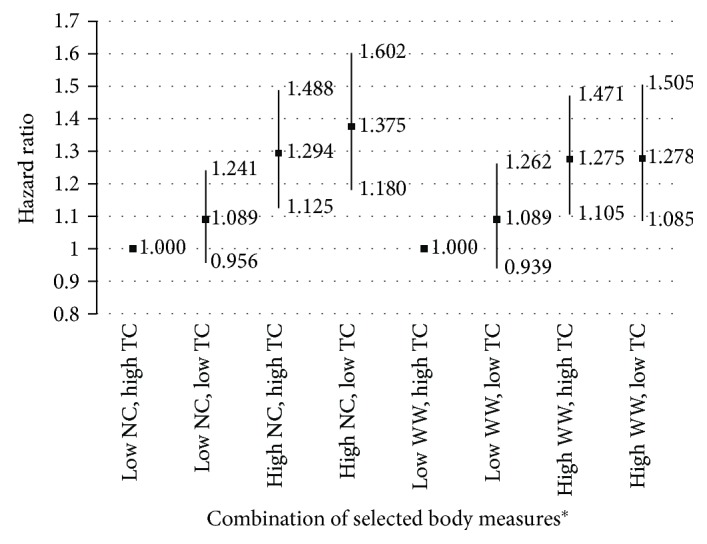
Association between body measurement combinations and HRs of T2DM occurrence. ^∗^NC: neck circumference; WW: waist width; TC: thigh circumference. Multivariable-adjusted HRs performed by Cox proportional hazard model adjusted for age, sex, education, marital status, occupation, betel nut chewing, and hypertension history.

The results of almost all selected body measurements were statistically significant between cases and controls. In general, T2DM cases had larger body measurements than controls (Table 2). Multivariable analyses indicated that NC (HR = 1.05; 95% CI = 1.03–1.06), WW (HR = 1.06; 95% CI = 1.04–1.08), and left TC (HR = 0.984; 95% CI = 0.972–0.995) were significantly associated with the occurrence of T2DM when adjusted for age, sex, education, marital status, occupation, betel nut chewing, and personal hypertension disease history (Table 3). The three body measurements were then further categorized into median high and median low groups, and combinations of NC/left TC and WW/left TC were made to further explore an association with high-risk groups. The results demonstrated a monotonic trend in multivariable-adjusted HRs for lower NC/lower TC (HR = 1.09; 95% CI = 0.96–1.24), higher NC/higher TC (HR = 1.29; 95% CI = 1.13–1.49), and higher NC/lower TC (HR = 1.38; 95% CI = 1.18–1.60), compared with lower NC/higher TC. The combination of WW and left TC followed a dose-dependent relationship according to lower WW/higher TC (HR = 1.00), lower WW/lower TC (HR = 1.09; 95% CI = 0.94–1.26), higher WW/higher TC (HR = 1.28; 95% CI = 1.11–1.47), and higher WW/lower TC (HR = 1.28; 95% CI = 1.09–1.51) (Figure 1).

## 4. Discussion

The results of this prospective cohort analysis indicate that NC, WW, and TC measurements can be used as alternative biomarkers for predicting the incidence of T2DM in the Taiwanese population. In addition to waist measurements, this study demonstrates that NC, TC, and NC/TC ratio are good independent predictors of the occurrence of T2DM (Appendix A (available here)). We did analyses on models with both prevalent and incident T2DM cases and only incident T2DM cases to ascertain the reliability of selected body measures and found NC, WW, and TC to be consistency predictors to T2DM. During the analyses, our study finds that NC has a positive correlation with WC (*r* = 0.61; *p* < 0.0001) and BMI (*r* = 0.59; *p* < 0.0001) and exhibits a strong association with the occurrence of T2DM. It is considered that NC measurements could be used as an alternative biomarker to WC in predicting T2DM. However, WW could be used as a supplementary measurement to NC, as it reflects the visceral fat capacity. Although the literature indicates that TC is negatively associated with the prevalence of T2DM, it was confirmed as a long-term predictor of the occurrence of T2DM in this study [20]. The present study proposes using a combination of neck, waist, and thigh measurements as reliable biomarkers for predicting the occurrence of T2DM.

NC has been positively and significantly associated with higher age, weight, BMI, WC, and hip circumference in both men and women [12–14]. In addition, NC may present as an alternative predictive factor for being overweight and obese and is correlated with central obesity. A larger NC has also been associated with a higher WC and values and levels of metabolic syndrome components, including SBP, DBP, dyslipidemia, and hyperglycemia [14–16]. Studies have demonstrated that NC is positively associated with the incidence of T2DM [24, 25] and that a larger NC has been associated with increased insulin resistance and various cardiometabolic risk factors, such as decreasing HDL levels and increasing plasma triglyceride levels among healthy Chinese adults [26]. Notably, insulin resistance is the core etiology of metabolic syndromes and is one of the principal pathogeneses of T2DM. Another study reported that NC may well predict the occurrence of gestational diabetes in Chinese women [27].

In this study, further evidence was found in the literature to support the association between NC and T2DM. A previous study proposed that using NC as a proxy indicator of upper-body fat may be superior to using it as a proxy indicator of whole-body fat in reflecting ectopic fat deposition and indicating cardiometabolic risk [9]. The measurement has also been shown to have a higher association with HOMA, low HDL-C, and triglycerides, compared with the use of WC [28]. Overall, NC is documented as a marker of upper-body subcutaneous adipose tissue distribution and a risk indicator of insulin resistance that may result in hyperglycemia and subsequent diabetes mellitus. Because it is easy to obtain, NC is becoming an alternative measurement; it not only exhibits a strong correlation with other body markers (such as WHR and BMI) but also is associated with physiological and biochemical measurements [29]. It is thus feasible for use as an innovative measurement in clinical practice and epidemiological surveys in large population communities.

Studies have indicated that larger hip and thigh circumferences are associated with a lower risk of T2DM, independent of age, BMI, and WC [28, 30]. The soft tissue in the thigh is mostly composed of muscle mass and subcutaneous fat. The skeletal muscles are the key target organ for insulin action and the site of insulin resistance [31]. Insulin resistance, the pathogenesis of T2DM, may interfere with glucose regulation and homeostasis, eventually resulting in hyperglycemia and leading to diabetes. Because a larger TC is related to a greater area of muscle mass, it is associated with a lower risk of diabetes [32]. A larger TC indicates not only a larger muscle mass but also an increased femoral subcutaneous fat mass. This is highly beneficial for the muscles and the liver and is free from high exposure to free fatty acid uptake and storage, thereby providing better insulin sensitivity and a superior lipid profile [33]. A lower muscle mass and less subcutaneous fat in the thighs may be associated with insulin resistance and eventually result in hyperglycemia and diabetes [19].

Our study also differs from those using traditional measurements because we used a combination of the NC/TC and WW/TC ratios to compare the HRs of T2DM incidence. These ratios may be useful as future screening measurements for identifying individuals at a high risk for diabetes. In our cohort study, a small NC and larger TC had a negative relationship with the incidence of diabetes. In addition, a small TC, in addition to being associated with diabetes, was associated with the development of heart disease or premature death [34].

WC and WHR are frequently used in current practice to estimate body fat accumulation on the trunk, and they represent an acknowledged risk factor for many metabolic disorders. The WHR offers a measurement of the relative accumulation of abdominal fat compared with the size of the body and is more widely adopted than using WC alone. Traditionally, WC has been a popular and useful body index for evaluating whether an individual was overweight and obese. One study showed that WC explains obesity-related health risk better than BMI and that for a given WC value, overweight, obese, and normal-weight persons have comparable health risks [35]. In this study, however, NC might be an alternative measure of a certain part of visceral fat and could provide a supplement to more comprehensive amount of body fat on the upper body. In addition, WC was replaced by WW as an indicator of the trunk's capacity to accumulate abdominal fat. Therefore, using a combination of WW and NC may offer a better indicator of upper-body fat accumulation in relation to subsequent long-term adverse effects.

Studies have demonstrated that other parameters, such as body weight, BMI, WC, and WHR, are associated with T2DM [5]. However, in this cohort study, NC, TC, and WW were the most significant predictors of T2DM in an Asian population. Many studies have shown that BMI is associated with obesity and metabolic syndrome, but the association is varied across ethnic groups [36–38]. The use of BMI to indicate body fat distribution is less reliable when used as an anthropometric index to predict adverse effects due to its inability to sufficiently relate to visceral fat. The WHR is useful to determine body size or muscular mass (using the hip circumference). However, it has been criticized because it can be biased by pelvic skeletal structure and width.

This study proposes a combination of NC and WW corrected using TC, as superior, comprehensive, and feasible measurements that can be used to predict future disease, such as insulin resistance and T2DM. Anthropometry is a feasible method that has a well-documented association with body fat distribution and metabolic complications. It is also cheap and easy to practice [39–41] in large population surveys and provides a reliable predictor of an individual's risk of adverse outcomes. Therefore, the measurements proposed in this study could be extended into both clinical practice and preventive medicine in the future.

To increase the accuracy of disease diagnosis, this study used accurate measuring techniques such as comprehensive whole-body scanning, computer-based technology (which reduced measurement errors), a longitudinal cohort analysis (which avoided temporal ambiguity), and national health insurance data tracking. Nevertheless, this study contains certain limitations. First, accurate radiographic measurements were not used to identify and quantify fat deposits; therefore, this study was unable to differentiate between fat tissue and muscular tissue using surface scanning. Secondly, the findings were derived from an Asian population, which serves inferences to the billions of people in Asia (such as in China), but generalizing to Western populations should be done with caution. Thirdly, there are confounders related to the information obtained in relation to the questionnaire and medical records. Some factors such as health-seeking behaviour, psychological measurements, and health knowledge were not determined, but nondifferential misclassification was assumed in this study. Finally, only one-shot measurements of 3D whole-body scanning were obtained, and therefore a study could be designed in the future that employs repeated measurements or records body changes.

## 5. Conclusions

NC, WW, and TC were found to be comprehensive and innovative measurements for use in independently predicting the occurrence of T2DM over the long term. A combination of NC and WW corrected by TC could be useful measurements for predicting an individual's future risk of developing T2DM. The body measurements shown in this study provide mechanistic conjectures for the early screening and diagnosis of T2DM in clinical practice.

## Figures and Tables

**Table 1:** Basic demographics, lifestyle variables, and personal and family disease history between study groups.

	Total (%)	Person-years	Number of T2DM	Incidence density (year^−1^)	Hazard ratio (HR)	95% CI
*Demographics*						
Age group	51.1 ± 11.9^∗^ (18.0, 90.7)	74964.5	2068	0.027586		
≤50.7275	4229 (50.1%)	39576.2	747	0.018875	1.000	—
>50.7275	4221 (50.0%)	35388.3	1321	0.037329	1.976	(1.806, 2.161)
Sex						
Female	4019 (47.6%)	36696.7	1030	0.028068	1.000	—
Male	4431 (52.4%)	38267.8	1038	0.027125	0.971	(0.891, 1.058)
Education						
Junior high school & below	3244 (38.4%)	27775.2	1011	0.036399	1.000	—
Senior high school	1719 (20.3%)	15,413	357	0.023162	0.639	(0.567, 0.721)
College/university & above	2813 (33.3%)	25843.9	528	0.020430	0.562	(0.506, 0.625)
Unknown	674 (8.0%)	5932.49	172	0.028993	0.798	(0.679, 0.938)
Marital status						
Married	6448 (76.3%)	57,660	1573	0.027281	1.000	—
Unmarried	1354 (16.0%)	11633.3	315	0.027077	0.994	(0.880, 1.122)
Unknown	648 (7.7%)	5671.25	180	0.031739	1.164	(0.997, 1.358)
Occupation						
Government	1169 (13.8%)	11724.5	291	0.024820	1.000	—
Farmers	587 (7.0%)	4745.08	160	0.033719	1.373	(1.132, 1.665)
Labourers	1065 (12.6%)	9581.96	199	0.020768	0.852	(0.711, 1.020)
Business	2559 (30.3%)	22030.3	555	0.025193	1.034	(0.897, 1.193)
Housekeeping & others	1933 (22.9%)	16640.2	575	0.034555	1.411	(1.225, 1.625)
Unknown	1137 (13.5%)	10242.4	288	0.028118	1.146	(0.974, 1.349)
*Lifestyle variables*						
Cigarette smoking						
No	6301 (74.6%)	56419.3	1553	0.027526	1.000	—
Yes	2149 (25.4%)	18545.2	515	0.027770	1.011	(0.915, 1.117)
Alcohol drinking						
No	6147 (72.8%)	54,897	1518	0.027652	1.000	—
Yes	2303 (27.3%)	20067.5	550	0.027407	0.994	(0.902, 1.096)
Betel nut chewing						
No	7826 (92.6%)	69575.5	1884	0.027078	1.000	—
Yes	624 (7.4%)	5388.99	184	0.034144	1.261	(1.084, 1.467)
*Underlying diseases*						
Hypertension						
No	6895 (81.6%)	62848.7	1515	0.024105	1.000	—
Yes	1423 (16.8%)	11150.4	531	0.047622	1.976	(1.790, 2.182)
Unknown	132 (1.6%)	965.437	22	0.022788	0.974	(0.639, 1.484)
Heart disease						
No	7700 (91.1%)	68690.6	1847	0.026889	1.000	—
Yes	618 (7.3%)	5308.45	199	0.037487	1.391	(1.202, 1.611)
Unknown	132 (1.6%)	965.437	22	0.022788	0.87	(0.571, 1.325)
*Family disease history*						
Hypertension						
No	5515 (65.3%)	49469.2	1333	0.026946	1.000	—
Yes	2845 (33.7%)	24865.1	721	0.028996	1.078	(0.984, 1.180)
Unknown	90 (1.1%)	630.259	14	0.022213	0.847	(0.500, 1.435)
Diabetes						
No	6613 (78.3%)	59774.9	1515	0.025345	1.000	—
Yes	1747 (20.7%)	14555.9	539	0.037030	1.466	(1.329, 1.617)
Unknown	90 (1.1%)	633.752	14	0.022091	0.897	(0.530, 1.520)
Stroke						
No	7197 (85.2%)	63978.1	1745	0.027275	1.000	—
Yes	1164 (13.8%)	10362.1	309	0.029820	1.093	(0.968, 1.233)
Unknown	89 (1.1%)	624.331	14	0.022424	0.844	(0.499, 1.429)
Heart disease						
No	7304 (86.4%)	64926.4	1783	0.027462	1.000	—
Yes	1056 (12.5%)	9403.82	271	0.028818	1.049	(0.923, 1.192)
Unknown	90 (1.1%)	634.366	14	0.022069	0.825	(0.488, 1.397)

^∗^Mean ± SD (min, max).

**Table 2:** Three-dimensional body surface measurements of T2DM cases and controls.

	Control	T2DM	*p* value
	Mean ± SD	Mean ± SD	
*Whole body*			
Height (cm)	161.4 ± 8.6	159.9 ± 8.5	<0.0001
Weight (kg)	63.9 ± 11.5	66.8 ± 12.1	<0.0001
Body mass index	24.4 ± 3.4	26.0 ± 3.6	<0.0001
*Head & neck*			
Head circumference (cm)	58.9 ± 2.0	58.9 ± 1.9	0.3969
Head surface area (cm^2^)	1337.3 ± 203.5	1313.0 ± 223.3	<0.0001
Head volume (cm^3^)	4720.5 ± 531.4	4765.7 ± 536.5	0.0016
Neck circumference (cm)	36.9 ± 4.0	37.9 ± 4.1	<0.0001
*Trunk*			
*Chest*			
Chest circumference (cm)	95.5 ± 9.2	99.2 ± 9.2	<0.0001
Waist circumference (cm)	86.3 ± 10.3	91.0 ± 11.0	<0.0001
Chest width (cm)	31.9 ± 2.8	32.4 ± 2.9	<0.0001
*Waist*			
Waist width (cm)	30.2 ± 3.1	31.2 ± 3.2	<0.0001
Chest sectional area (cm^2^)	636.0 ± 121.1	689.7 ± 126.3	<0.0001
Waist sectional area (cm^2^)	568.7 ± 137.6	632.5 ± 150.5	<0.0001
Trunk surface area (cm^2^)	6646.4 ± 1642.4	6712.4 ± 1928.5	0.1875
Trunk volume (cm^3^)	37859.3 ± 7921.9	40685.6 ± 8550.9	<0.0001
*Upper limbs*			
Arm length (cm)			
Right	56.4 ± 3.5	56.1 ± 3.4	0.0112
Left	55.8 ± 3.5	55.5 ± 3.4	0.0028
Upper arm circumference (cm)			
Right	29.8 ± 3.8	31.0 ± 4.0	<0.0001
Left	30.2 ± 4.0	31.3 ± 4.2	<0.0001
Forearm circumference (cm)			
Right	24.2 ± 2.5	24.9 ± 2.7	<0.0001
Left	24.4 ± 2.8	24.9 ± 2.8	<0.0001
Arm surface area (cm^2^)			
Right	1512.8 ± 275.5	1487.9 ± 259.0	0.0002
Left	1526.5 ± 316.8	1487.8 ± 297.8	<0.0001
Arm volume (cm^3^)			
Right	2338.2 ± 479.0	2421.9 ± 478.3	<0.0001
Left	2392.4 ± 544.4	2437.3 ± 541.8	0.0011
*Hip*			
Hip circumference (cm)	96.6 ± 6.2	98.5 ± 6.6	<0.0001
Hip width (cm)	34.3 ± 2.5	34.8 ± 2.4	<0.0001
Hip sectional area (cm^2^)	675.4 ± 105.1	711.9 ± 110.7	<0.0001
*Lower limbs*			
Leg length (cm)			
Right	68.8 ± 5.0	67.7 ± 5.0	<0.0001
Left	68.8 ± 5.0	67.7 ± 5.0.	<0.0001
Thigh circumference (cm)			
Right	50.9 ± 4.9	52.0 ± 5.0	<0.0001
Left	50.8 ± 5.0	52.0 ± 5.1	<0.0001
Calf circumference (cm)			
Right	33.6 ± 3.5	34.0 ± 3.4	<0.0001
Left	33.5 ± 3.5	34.0 ± 3.5	<0.0001
Leg surface area (cm^2^)			
Right	2282.3 ± 415.4	2246.4 ± 420.3	0.0007
Left	2244.8 ± 378.4	2214.5 ± 364.4	0.0011
Leg volume (cm^3^)			
Right	6035.9 ± 1373.0	6166.2 ± 1370.1	0.0002
Left	5990.5 ± 1373.6	6141.9 ± 1368.3	<0.0001

**Table 3:** Factors associated with the occurrence of T2DM by multiple Cox regression analysis.

	HR	95% CI	*p* value
Age			
≤50.7275	1.000	—	—
>50.7275	1.521	(1.364, 1.695)	<0.0001
Sex			
Female	1.000	—	—
Male	0.727	(0.639, 0.827)	<0.0001
Education			
Junior high school & below	1.000	—	—
Senior high School	0.835	(0.727, 0.958)	0.0101
College/university & above	0.711	(0.617, 0.819)	<.0001
Unknown	0.872	(0.712, 1.069)	0.1874
Marital status			
Married	1.000	—	—
Unmarried	1.038	(0.911, 1.183)	0.5734
Unknown	1.163	(0.952, 1.420)	0.1395
Occupation			
Government	1.000	—	—
Farmers	0.821	(0.654, 1.030)	0.0877
Labourers	0.730	(0.595, 0.896)	0.0026
Business persons	0.847	(0.718, 1.000)	0.0496
Housewives/husbands & other categories	0.854	(0.713, 1.022)	0.0853
Unknown	0.766	(0.624, 0.939)	0.0102
Betel nut chewing			
No	1.000	—	—
Yes	1.291	(1.093, 1.525)	0.0027
Neck circumference	1.048	(1.033, 1.064)	<.0001
Waist width	1.061	(1.040, 1.081)	<.0001
Left thigh circumference	0.984	(0.972, 0.995)	0.0055
Right upper arm circumference	1.030	(1.016, 1.045)	<0.0001
Hypertension			
No	1.000	—	—
Yes	1.466	(1.315, 1.633)	<0.0001
Unknown	0.770	(0.494, 1.200)	0.2482

## Supplementary Materials

Supplementary Materials Supplementary Material Appendix A: incremental model goodness of fit of different body measures.
