# Supplementary material for: Predicting Type 2 Diabetes Mellitus Occurrence Using Three-Dimensional Anthropometric Body Surface Scanning Measurements: A Prospective Cohort Study
Source: J Diabetes Res. 2018 Jul 8;2018:6742384. doi: 10.1155/2018/6742384 (PMC6079414; doi:10.1155/2018/6742384)
Supplement: Supplementary Materials — Supplementary Material Appendix A: incremental model goodness of fit of different body measures. [file 6742384.f1.pdf]

Appendix A. Incremental model goodness of fit of different body measures

Measures	Waist circumference	Waist/hip ratio	NC, WW, TC	NC/TC ratio	WW/TC ratio
Difference of -2 Log likelihood*	250.99	402.56	3551.41	3410.95	47.53

NC=neck circumference; TC=left thigh circumference; WW=waist width; waist/hip ratio=waist circumference/hip circumference

* Differences between model goodness of fit value in terms of -2 Log likelihood of the basic model and of the model added with test body measures. The basic model adjusted for age, sex, education, marital status, occupation, betel nut chewing, and hypertension history.
